# Genetic variants of *CYP3A5*, *CYP2D6*, *SULT1A1*, *UGT2B15 *and tamoxifen response in postmenopausal patients with breast cancer

**DOI:** 10.1186/bcr1640

**Published:** 2007-01-23

**Authors:** Pia Wegman, Sauli Elingarami, John Carstensen, Olle Stål, Bo Nordenskjöld, Sten Wingren

**Affiliations:** 1 Department of Biomedicine and Surgery, Division of Cell Biology, Faculty of Health Sciences, Linköping University, 581 85 Linköping, Sweden; 2 Department of Health and Society, Faculty of Arts and Sciences, Linköping University, 581 83 Linköping, Sweden; 3 Department of Biomedicine and Surgery, Division of Oncology, Faculty of Health Sciences, Linköping University, 581 85 Linköping, Sweden

## Abstract

**Introduction:**

Tamoxifen therapy reduces the risk of recurrence and prolongs the survival of oestrogen-receptor-positive patients with breast cancer. Even if most patients benefit from tamoxifen, many breast tumours either fail to respond or become resistant. Because tamoxifen is extensively metabolised by polymorphic enzymes, one proposed mechanism underlying the resistance is altered metabolism. In the present study we investigated the prognostic and/or predictive value of functional polymorphisms in cytochrome P450 3A5 CYP3A5 (*3), CYP2D6 (*4), sulphotransferase 1A1 (SULT1A1; *2) and UDP-glucuronosyltransferase 2B15 (UGT2B15; *2) in tamoxifen-treated patients with breast cancer.

**Methods:**

In all, 677 tamoxifen-treated postmenopausal patients with breast cancer, of whom 238 were randomised to either 2 or 5 years of tamoxifen, were genotyped by using PCR with restriction fragment length polymorphism or PCR with denaturing high-performance liquid chromatography.

**Results:**

The prognostic evaluation performed in the total population revealed a significantly better disease-free survival in patients homozygous for CYP2D6*4. For *CYP3A5*, *SULT1A1 *and *UGT2B15 *no prognostic significance was observed. In the randomised group we found that for *CYP3A5*, homozygous carriers of the *3 allele tended to have an increased risk of recurrence when treated for 2 years with tamoxifen, although this was not statistically significant (hazard ratio (HR) = 2.84, 95% confidence interval (CI) = 0.68 to 11.99, *P *= 0.15). In the group randomised to 5 years' tamoxifen the survival pattern shifted towards a significantly improved recurrence-free survival (RFS) among CYP3A5*3-homozygous patients (HR = 0.20, 95% CI = 0.07 to 0.55, *P *= 0.002). No reliable differences could be seen between treatment duration and the genotypes of *CYP2D6*, *SULT1A1 *or *UGT2B15*. The significantly improved RFS with prolonged tamoxifen treatment in CYP3A5*3 homozygotes was also seen in a multivariate Cox model (HR = 0.13, CI = 0.02 to 0.86, *P *= 0.03), whereas no differences could be seen for *CYP2D6*, *SULT1A1 *and *UGT2B15*.

**Conclusion:**

The metabolism of tamoxifen is complex and the mechanisms responsible for the resistance are unlikely to be explained by a single polymorphism; instead it is a combination of several mechanisms. However, the present data suggest that genetic variation in *CYP3A5 *may predict response to tamoxifen therapy.

## Introduction

Tamoxifen is widely used as an endocrine treatment for patients with oestrogen-receptor (ER)-positive breast cancer. Five years of adjuvant tamoxifen therapy reduces the risk of recurrence and prolongs the survival of women with ER-positive tumours [1]. Nevertheless, in a proportion of patients, tumours are resistant to tamoxifen, or become so, with a subsequent relapse in the disease. The mechanisms underlying the resistance are not fully understood. Because there is convincing evidence that tamoxifen is converted to anti-oestrogenic metabolites that are more potent than the mother substance, one hypothesis is that altered metabolism might contribute to inter-individual variability in serum concentrations, which in turn may influence the action on ER and the response to treatment [2-4]. Some studies have demonstrated considerable inter-individual variation in concentrations of tamoxifen metabolites both in plasma and locally in the breast [5,6].

Several primary metabolites have been identified, including *N*-desmethyl-tamoxifen, 4-hydroxy-*N*-desmethyl-tamoxifen (endoxifen) and 4-hydroxy-tamoxifen. Whereas tamoxifen has a relatively low affinity for ER, the metabolite 4-hydroxy-tamoxifen binds with an affinity similar to that of oestradiol [7,8]. It has also been demonstrated by studies *in vitro *that endoxifen exhibits a cpotency similar to that of 4-hydroxy-tamoxifen with regard to ER binding affinity, suppression of oestrogen-dependent cell growth and gene expression [2-4]. *N*-desmethyl-tamoxifen, in contrast, is a weak anti-oestrogen [2,3,7-9]. Previous studies have shown that cytochrome P450 3A4 and cytochrome P450 3A5 (CYP3A4 and CYP3A5) are the major catalysts of *N*-demethylation, whereas the 4-hydroxylation is predominantly supported by the cytochrome P450 2D6 (CYP2D6) [10-12]. The 4-hydroxy-tamoxifen is in equilibrium between a *trans *and a *cis *isoform, resulting in a proposed shift in property from a potent anti-oestrogen towards a significantly less potent one [13-15]. *Cis*-4-hydroxy-tamoxifen has in addition been reported to exist at higher concentrations than *trans*-4-hydroxy-tamoxifen in patients with breast cancer with acquired resistance to tamoxifen [16]. Nishiyama and colleagues [17] suggested a geometrical selectivity in both the sulphation of *trans*-4-hydroxy-tamoxifen and the glucuronidation of *cis*-4-hydroxy-tamoxifen, and that these reactions were catalysed by sulphotransferase 1A1 (SULT1A1) and UDP-glucuronosyltransferase 2B15 (UGT2B15), respectively [17].

Polymorphisms affecting enzyme activity have been found in CYP3A5, CYP2D6, SULT1A1 and UGT2B15. The most frequent and functionally important polymorphism in the *CYP3A5 *gene consists of an A6986G transition within intron 3 (CYP3A5*3). This polymorphism creates an alternative splice site resulting in a frame shift and truncation of the protein [18-20]. In *CYP2D6 *the most common non-functional allele is CYP2D6*4. This polymorphism generates a G→A transition at the first nucleotide of exon 4 in the *CYP2D6 *gene, leading to a truncated non-functional gene product [21]. Further, the most frequent polymorphism in the *SULT1A1 *gene is a G→A transition at nucleotide 638, defining the SULT1A1*2 allele, which is correlated with a diminished capacity to sulphate SULT1A1 substrates [22]. Several polymorphisms have also been found within the *UGT2B15 *gene although only two in the coding region [23]. One causes an amino acid shift from aspartate (UGT2B15*1) to tyrosine (UGT2B15*2) at position 85 of the UGT2B15 protein, a shift that has been associated with an increase in enzyme activity [24]. In a previous study we investigated functional polymorphisms in *CYP2D6 *and *SULT1A1 *in 226 postmenopausal patients with breast cancer randomised to treatment with or without tamoxifen. We found a benefit of tamoxifen in carriers of the inactive allele CYP2D6*4 and a tendency in patients homozygous for SULT1A1*1, when compared with the control population [25]. In the present study a new cohort was investigated that was both larger and included additional polymorphic enzymes involved in the metabolism of tamoxifen. The polymorphisms of *CYP3A5 *(CYP3A5*1 and *3), *CYP2D6 *(CYP2D6*1 and *4), *SULT1A1 *(SULT1A1*1 and *2) and *UGT2B15 *(UGT2B15*1 and *2) were examined in tamoxifen-treated postmenopausal patients with breast cancer, to discern whether the genotypes correlated with clinical/pathological factors and/or to the benefit of tamoxifen. We proposed that the alleles contributing to the bioactivation of tamoxifen (namely CYP3A5*1 and CYP2D6*1) as well as the alleles delaying the elimination of active metabolites (namely SULT1A1*2 and UGT2B15*1) would be favourable.

## Materials and methods

### Patients

We analysed frozen tumour tissues from 677 postmenopausal patients with stage II and III breast cancer. They were diagnosed between 1986 and 1997 in the South East Health Care Region of Sweden and were between 50 and 96 years old, with a mean age of 69 years at the time of diagnosis. Information on tumour size, lymph-node status and tumour stage is shown in Table 1. All patients were ER-positive and had received adjuvant postoperative tamoxifen therapy. A subgroup of 238 patients was randomised to either 2 or 5 years of treatment with tamoxifen. Patients diagnosed before 1994 received a daily dosage of 40 mg tamoxifen for 2 or 5 years. After 1 January 1994 all postmenopausal patients received 20 mg for 5 years. In the non-randomised group (*n *= 439) 175 patients were treated with 40 mg and 264 patients with 20 mg of tamoxifen. The mean follow-up time in the total population was 7.3 years (range 0.04 to 17.9, median 7.08). For the randomised group the mean follow-up time in the 2-year arm was 9.8 years (range 2.3 to 17.7, median 9.8) and in the 5-year arm 10.7 years (range 2.1 to 17.9, median 10.7). The study was approved by the local ethical committee in Linköping, Sweden (register number 03-608).

### DNA isolation and PCR

Fresh frozen breast tumour tissues (about 30 mg) were homogenised with a microdismembrator (B Braun, Melsungen, Germany), and genomic DNA was then isolated with the SV Genomic DNA Purification System (Promega Corporation, Madison, WI, USA). The DNA content was determined by spectrophotometry, and purified DNA was stored at -20°C before use.

The *CYP3A5*, *CYP2D6*, *SULT1A1 *and *UGT2B15 *genes were amplified by PCR in separate reactions with 50 ng of DNA. The primer sequences used in the PCR of *CYP2D6 *and *SULT1A1 *were adopted from Hanioka and colleagues [21] and Coughtrie and colleagues [26], respectively, whereas the *CYP3A5 *and *UGT2B15 *genes were amplified with forward (5'-ACCACCCAGCTTAACGAATG-3' and 5'-AGAGCTTGTTCAGAGGGGTCATGAG-3') and reverse (5'-AGCACAGGGAGTTGACCTTC-3' and 5'-AAATTCTCGATAGATGGATATATGG-3') primers, respectively. The following PCR reagents were added to a reaction volume of 20 μl: 2 mM MgCl_2_, 0.2 mM dNTPs, 0.5 unit of *Taq *DNA polymerase, and 1 μM each of forward and reverse primer in 1 × PCR buffer. The amplification was performed in a PTC-200 Peltier Thermal Cycler DNA Engine (MJ Research™ Inc, Waltham, MA, USA). An initial denaturation at 94°C for 3 minutes was followed by 38 to 40 cycles of 30 seconds at 94°C, 30 seconds of annealing at 63°C (*CYP2D6*, *SULT1A1*) or 64°C (*CYP3A5*, *UGT2B15*), and 40 seconds of extension at 72°C. An extension period of 5 minutes followed the final cycle. Fragments were then resolved by electrophoresis on a 2% (w/v) agarose gel containing 1 × TBE (89 mM Tris, 89 mM boric acid, 2 mM EDTA, pH 8.4) buffer and ethidium bromide (0.5 μg/μl). The gel was finally processed in an ultraviolet detector (Spectromics Corporation, New York, NY, USA).

### Restriction fragment length polymorphism

The *CYP2D6 *and *SULT1A1 *polymorphisms were detected by using restriction enzymes. The *Mva*I restriction enzyme distinguishes between the CYP2D6*4 and other *CYP2D6 *alleles. The polymorphic allele CYP2D6*4 lacks the restriction site and is thereby retained as one fragment. The wild-type allele SULT1A1*1 has a restriction site recognised by *Hae*II but the polymorphic allele SULT1A1*2 does not. *Mva*I (10 units; Fermentas, Stockholm, Sweden) and 1.5 μl of R^+ ^buffer (supplied by the manufacturer) were added to each tube of *CYP2D6 *PCR products and incubated at 37°C for 2.5 hours. The *SULT1A1 *PCR products were incubated with 5 units of the restriction enzyme *Hae*II (New England BioLabs, Beverly, MA, USA) in a 20 μl reaction mixture containing 1 × NE buffer (50 mM potassium acetate, 20 mM Tris-acetate, 10 mM magnesium acetate, 1 mM dithiothreitol, pH 7.9; New England BioLabs) supplemented with 100 μg/ml bovine serum albumin. After digestion, fragments were resolved by electrophoresis on a 3% (w/v) agarose gel containing 1 × TBE buffer and ethidium bromide (0.5 μg/μl). The gel was finally processed in an ultraviolet detector. To confirm the genotypes obtained with the restriction fragment length polymorphism method, 20 randomly selected samples were DNA sequenced and no differences in genotype were detected between the methods.

### Transgenomic Wave^® ^– nucleic acid fragment analysis system (denaturing high-performance liquid chromatography)

The Transgenomic Wave^® ^system is based on the principle of liquid chromatography and was used to separate and detect the CYP3A5*1, *3 and UGT2B15*1, *2 alleles amplified by PCR. In this system, DNA fragments are carried by a gradient buffer (with a 2% increase in buffer per minute) through a DNASep^® ^Cartridge under isothermal (58°C and 55.6°C, respectively) conditions, with subsequent detection by absorbance at 260 nm. The buffers used in the system consists of 0.1 M triethylammonium acetate (TEAA) and 25% (v/v) acetonitrile and 0.1 M TEAA (gradient buffer). To estimate the retention pattern for the *UGT2B15 *and *CYP3A5 *alleles, a few samples were DNA sequenced by a Thermo Sequenase Kit (Amersham Pharmacia Biotech, Uppsala, Sweden) and used as controls for each genotype.

### Statistical analyses

SPSS Advanced Models™ 12.0 software was used for the statistical analyses. The χ^2 ^test was used to show differences in the distribution of genotypes according to lymph-node status, tumour size and tumour stage. In the survival analyses, each woman's contribution to the person-years at risk began from the date of initial surgery to 1 January 2004, to the date of local or distant recurrences, whichever was earlier. The survival curves of recurrences were estimated with the Kaplan–Meier method, and the significance of differences between survival rates, for patients with different genotypes and endocrine treatment, was assessed by log-rank test. A univariate Cox proportional-hazard model was used for the estimation of the hazard ratio (HR) comparing genotype for each treatment group. Further, we performed a multivariate Cox model to adjust for the different tumour characteristics between the genotypes. We also tested whether the calculated hazard ratios were significantly different between treatment groups by using an interaction test according to the Cox model (treatment (0/1), genotype (0/1) and treatment × genotype (0/1)). Differences between groups were judged significant at confidence levels greater than 95% (*P *< 0.05).

## Results

The frequencies of the most common alleles were 0.92 (CYP3A5*3), 0.82 (CYP2D6*1), 0.61 (SULT1A1*1) and 0.52 (UGT2B15*2). Homozygous genotypes representing blocked or diminished enzymatic activity, CYP3A5*3, CYP2D6*4 and SULT1A1*2, were seen in 84.9%, 5.0% and 15.2% of the patients, respectively. Information on genotype and tumour characteristics is shown in Table 1. There was no significant difference in the distribution of tumour characteristics according to genotype.

**Figure 1:**
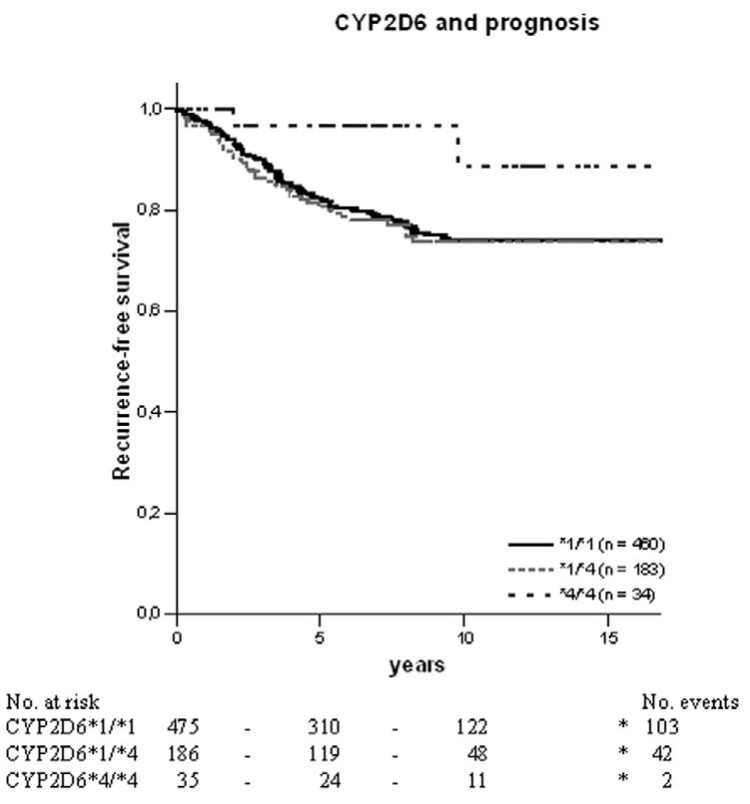
Kaplan–Meier estimates for recurrence-free survival between *CYP2D6 *genotypes in oestrogen-receptor-positive postmenopausal breast cancer patients. The black solid line represents patients homozygous for CYP2D6*1, the grey solid line represents heterozygous patients, and the dotted line represents patients homozygous for CYP2D6*4. *P *= 0.05 between *4/*4 and *1/*1; *P *= 0.04 between *4/*4 and *1/*4; *P *= 0.16 between *1/*1 and *1/*4.

To evaluate whether the genotypes were of prognostic importance in terms of recurrence-free survival (RFS), we calculated each polymorphism separately. The Kaplan–Meier estimates demonstrated that patients homozygous for the CYP2D6*4 allele had a significantly better prognosis than patients who were homozygous or heterozygous for the *1 allele (*P *= 0.05 and *P *= 0.04, respectively) (Figure 1). In a multivariate Cox analysis including tumour stage, tumour size and lymph-node status, the result for *CYP2D6 *was less clear (*P *= 0.055). For *CYP3A5*, *SULT1A1 *and *UGT2B15*, no significant difference was seen regarding prognosis (data not shown).

Among the 677 postmenopausal patients with breast cancer, a subgroup (*n *= 238) was randomised to either 2 or 5 years of adjuvant tamoxifen. In the randomised group we examined whether the genotypes of *CYP3A5*, *CYP2D6*, *SULT1A1 *and *UGT2B15 *could influence the benefit of tamoxifen. When calculating the predictive value, cases with less than 2 years of follow-up time were excluded. In the statistical analyses we combined homozygous and heterozygous patients to equalise the different groups when the number of either homozygous genotype was low. Initially, we compared the RFS by genotype and by randomisation with log-rank test. Next, we estimated the HR for each genotype in the two treatment groups by using a univariate Cox proportional hazard model. Finally, we performed a multivariate Cox model adjusted for tumour characteristics such as stage, size, lymph-node involvement and tamoxifen duration. The predictive value of each genotype was further evaluated with an interaction test that compared the estimated HRs.

**Figure 2:**
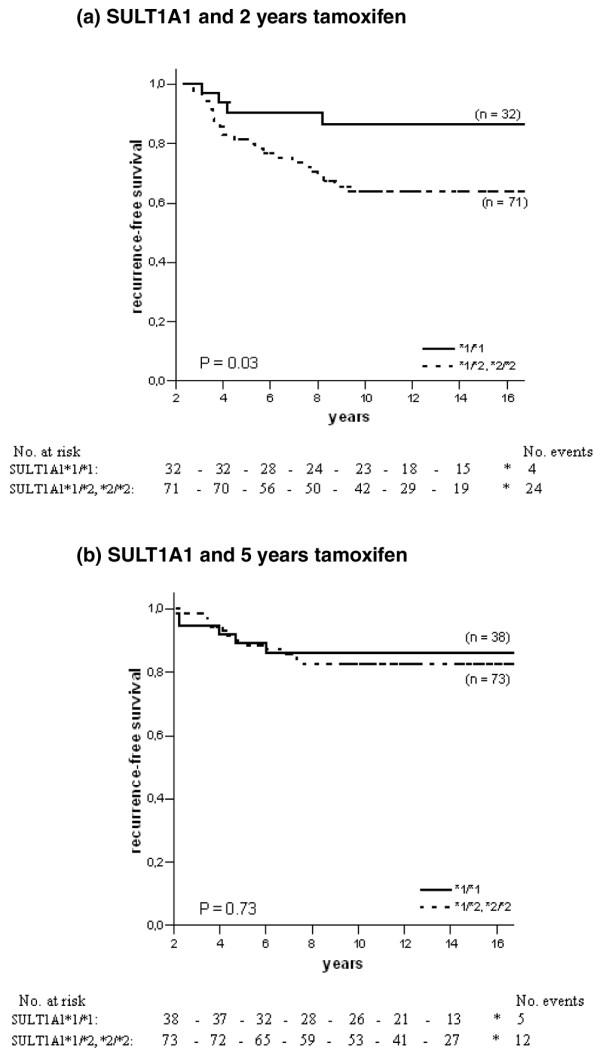
Recurrence-free survival in ER-positive patients with different genotypes of *SULT1A1 *and tamoxifen randomisation. The solid line represents patients homozygous for the SULT1A1*1 allele, and the dotted line represents patients homozygous or heterozygous for the SULT1A1*2 allele. **(a) ***SULT1A1 *and 2 years of tamoxifen therapy; **(b) ***SULT1A1 *and 5 years of tamoxifen therapy.

The HRs presented in Table 2 demonstrate significant associations for the *SULT1A1 *and *CYP3A5 *genotypes and RFS. We found an improved RFS with 2 years of tamoxifen in homozygous carriers of the SULT1A1*1 allele (HR = 0.33, 95% CI = 0.12 to 0.96, *P *= 0.04). However, no such difference was detected in patients randomised to 5 years of tamoxifen. The survival curves of *SULT1A1 *are shown in Figure 2.

**Figure 3:**
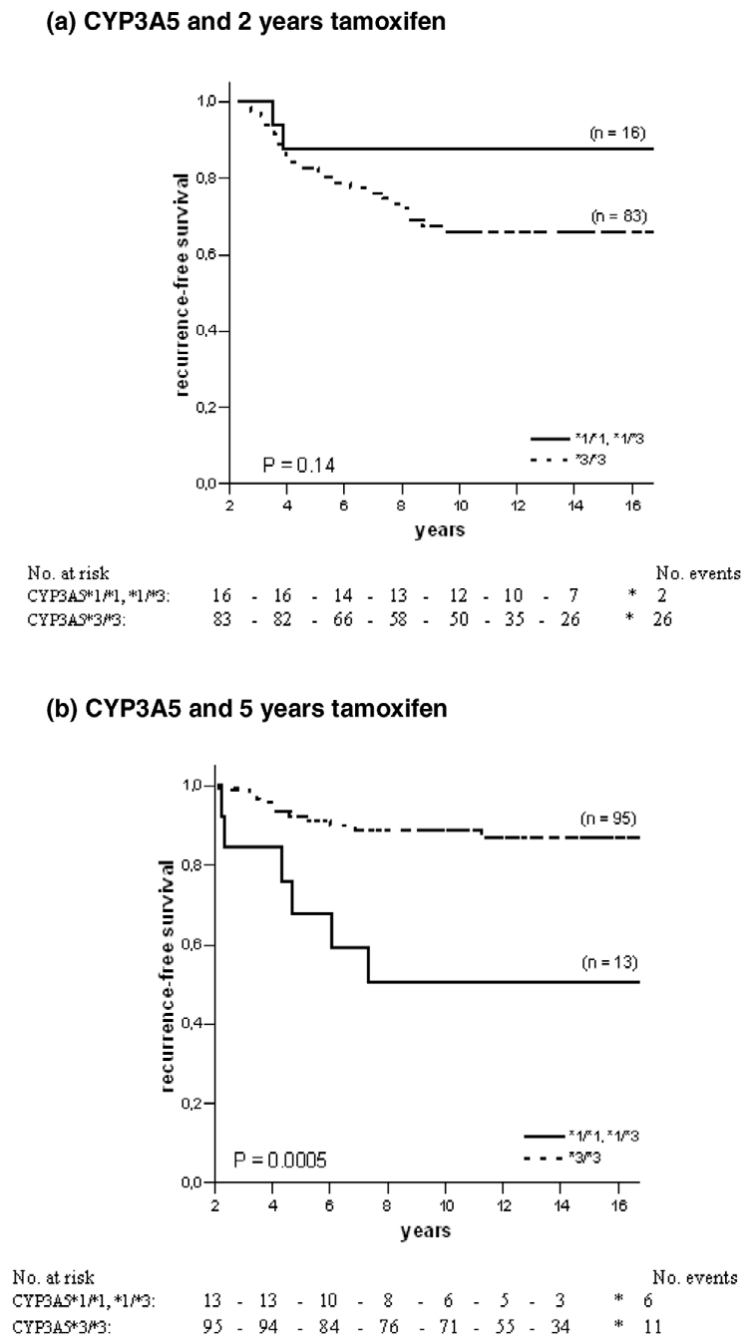
Recurrence-free survival in ER-positive patients with different genotypes of *CYP3A5 *and tamoxifen randomisation. The solid line represents patients homozygous or heterozygous for the CYP3A5*1 allele, and the dotted line represents patients homozygous for the CYP3A5*3 allele. **(a) ***CYP3A5 *and 2 years of tamoxifen therapy; **(b) ***CYP3A5 *and 5 years of tamoxifen therapy.

**Figure 4:**
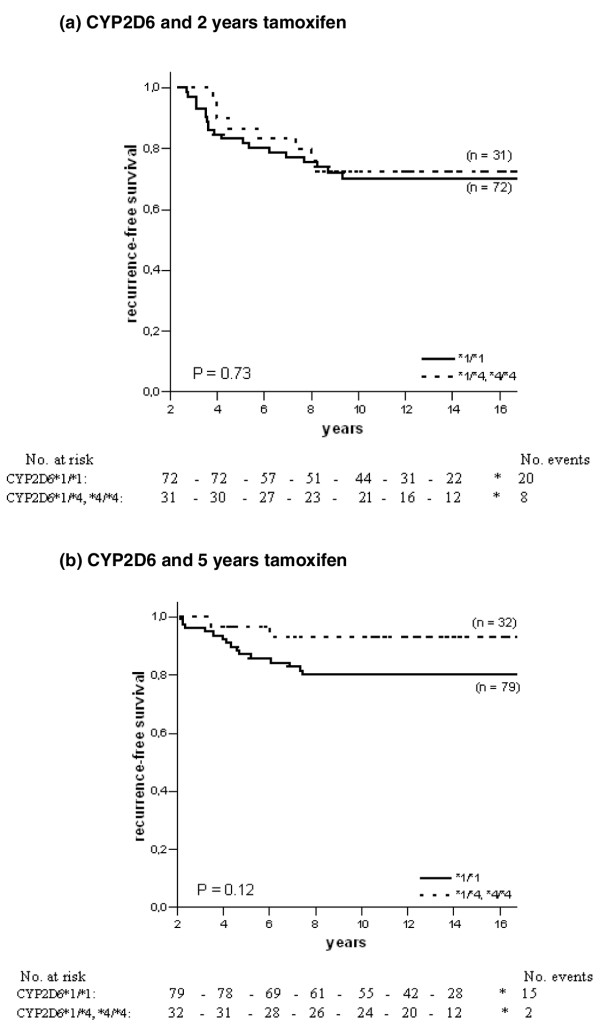
Recurrence-free survival in ER-positive patients with different genotypes of *CYP2D6 *and tamoxifen randomisation. The solid line represents patients homozygous for the CYP2D6*1 allele, and the dotted line represents patients homozygous or heterozygous for the CYP2D6*4 allele. **(a) ***CYP2D6 *and 2 years of tamoxifen therapy; **(b) ***CYP2D6 *and 5 years of tamoxifen therapy.

**Figure 5:**
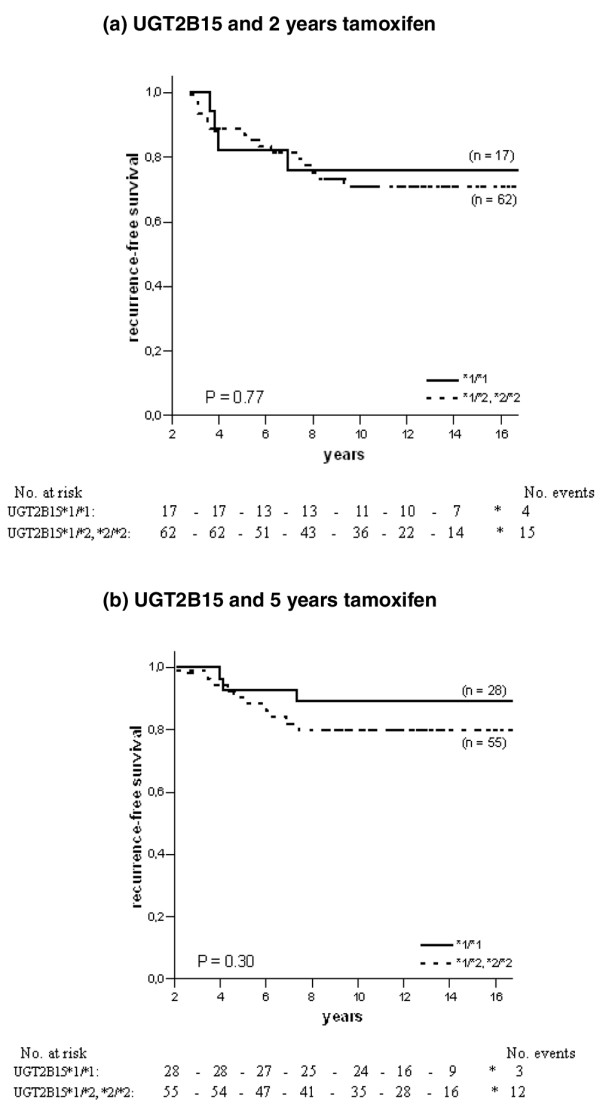
Recurrence-free survival in ER-positive patients with different genotypes of *UGT2B15 *and tamoxifen randomisation. The solid line represents patients homozygous for the UGT2B15*1 allele, and the dotted line represents patients homozygous or heterozygous for the UGT2B15*2 allele. **(a) ***UGT2B15 *and 2 years of tamoxifen therapy; **(b) ***UGT2B15 *and 5 years of tamoxifen therapy.

For *CYP3A5*, homozygous carriers of the *3 allele tended to have an increased risk of recurrence when treated with 2 years of tamoxifen, although this was not statistically significant (HR = 2.84, 95% CI = 0.68 to 11.99, *P *= 0.15; Table 2 and Figure 3a). In the group randomised to 5 years of tamoxifen the survival pattern shifted towards a significantly improved RFS among CYP3A5*3 homozygous patients (HR = 0.20, 95% CI = 0.07 to 0.55, *P *= 0.002; Table 2). The survival curve is shown in Figure 3b (*P *= 0.0005). When comparing HRs the benefit of 5 years of tamoxifen associated with homozygous carriers of the CYP3A5*3 allele persisted (*P *= 0.003; Table 2). The other genes examined (*CYP2D6 *and *UGT2B15*) showed no significant difference by genotype (Figures 4 and 5 and Table 2), even though patients homozygous or heterozygous for CYP2D6*4 had a tendency towards improved benefit of 5 years of tamoxifen compared with patients homozygous for CYP2D6*1 (*P *= 0.12). Furthermore, when we adjusted for tamoxifen duration, tumour stage, tumour size and lymph-node status and performed an interaction test when significance occurred, patients homozygous for CYP3A5*3 still had a significantly decreased risk of recurrence when treated for 5 years with tamoxifen (HR = 0.13, CI = 0.02 to 0.86, *P *= 0.03). No differences could be seen for *CYP2D6*, *SULT1A1 *and *UGT2B15*.

## Discussion

In a previous report of patients with breast cancer randomised to treatment with and without tamoxifen we found that genetic polymorphism in *CYP2D6 *and *SULT1A1 *may predict the benefit of tamoxifen therapy with a significantly improved disease-free survival in patients that were carriers of the CYP2D6*4 allele and/or were homozygous for the SULT1A1*1 allele [25]. In the present investigation we examined a different and larger cohort, which also included additional polymorphic enzymes that participate in the biotransformation and elimination of tamoxifen. The study design between these investigations differs, because all patients received tamoxifen in the current study. However, of the 677 patients with breast cancer analysed, a subgroup was randomised to either 2 or 5 years of tamoxifen and an attempt was made to calculate the predictive value of the polymorphic enzymes within the randomised population (*n *= 238). In this population, patients were treated differently 2 years after the start of tamoxifen treatment and we therefore compared RFS for each randomised group and genotype. In the group treated with 5 years of tamoxifen we found that patients homozygous for the CYP3A5*3 variant allele had a significantly improved RFS compared with patients harbouring the CYP3A5*1 allele, and we could also see a weak tendency for a decreased risk of recurrence in patients who were carriers of the CYP2D6*4 variant allele. However, the relevance of this finding is uncertain because the number of patients was limited.

A comparison of the hazard ratios calculated for each treatment group demonstrated that the risk reduction was significantly higher in patients homozygous for the CYP3A5*3 allele than in those harbouring the CYP3A5*1 allele. This was also true when we adjusted for tumour size, tumour stage and lymph-node status. No association between the outcome of tamoxifen treatment and the genotypes of *CYP2D6*, *SULT1A1 *and *UGT2B15 *was found. Even if the assessment of *CYP2D6 *showed no significant influence in the randomised groups, there was a tendency for an advantage with 5 years of tamoxifen in carriers of at least one CYP2D6*4 allele. This is partly in agreement with our previous findings [25] and with those of Nowell and colleagues [27], who noted that the CYP2D6*4 variant seemed to be associated with a decreased risk of death or recurrence. In contrast, a recent prognostic study by Goetz and colleagues [28] demonstrated that patients homozygous for CYP2D6*4 had both significantly worse relapse-free time and disease-free survival but not overall survival. Because their homozygous CYP2D6*4 genotype constitutes a small number of patients, results should therefore be confirmed in a larger patient population.

Several studies have been performed on the CYP3A5 enzyme and tamoxifen, although results are controversial. The reports have focused mainly on the concentration of metabolites and its association with genotype or patient outcome. Goetz and colleagues [28] attempted to investigate whether CYP3A5*3 polymorphism affected patient outcome. They found no differences in relapse-free time, disease-free survival or overall survival by CYP3A5*3 genotype. Our novel findings of an improved RFS in patients homozygous for CYP3A5*3 was unexpected because this genotype represents an inactive form of the enzyme and should therefore not catalyse the formation of the primary metabolite *N*-desmethyl-tamoxifen, which is a precursor of the ER-active metabolite endoxifen. Tucker and colleagues [29] recently tested whether genetic polymorphisms in *CYP3A5 *were associated with altered metabolism of tamoxifen in patients with breast cancer. They found no significant differences in tamoxifen or metabolite concentration by CYP3A5*3 polymorphic status. In contrast, Jin and colleagues [30] suggested that subjects who carried at least one CYP3A5*1 allele had higher plasma concentrations of endoxifen than those lacking functional *CYP3A5 *alleles (namely *3/*3), although this was not statistically significant. In addition, several authors have proposed that CYP3A5 might be a minor contributor to the overall metabolism of CYP3A substrates [31-33]. There is also a large overlap in substrate specificity between CYP3A4 and CYP3A5, so the contribution of each CYP3A4 and CYP3A5 to total CYP3A activity could thus depend on both the drug and the individual exposed to it [34].

The influence of *SULT1A1 *in the current investigation did not reveal any conclusive implication, either in the prognostic nor in the predictive evaluation. The SULT1A1*1 allele was previously investigated by Nowell and colleagues [35] and by our group [25]; both studies indicated that the high-activity allele SULT1A1*1 contributed significantly to tamoxifen response. In a more recent study by Nowell and colleagues [27], genetic polymorphism in *SULT1A1 *and *UGT2B15 *was analysed in patients with breast cancer treated with or without tamoxifen. They demonstrated that genetic variation in these phase II enzymes alone or in combination was associated with overall survival and recurrence of disease. Individuals possessing both UGT2B15*2 and SULT1A1*2 alleles had a significantly increased risk of death. *SULT1A1 *was also investigated by Jin and colleagues [30], who studied whether the mean plasma concentration of tamoxifen and its metabolites was associated with genetic variants of this gene, but no such correlation was found.

Co-administration of drugs (such as selective serotonin reuptake inhibitors (SSRI)) used in patients with breast cancer has been shown to inhibit the metabolism of tamoxifen [2,30]. This is an important issue because changes in the concentrations of active metabolites might influence the outcome of therapy; however, results are controversial [2,30,36,37]. The influence from SSRI co-administration in our investigation is likely to be a minor problem because such drugs were rarely used in the current study population.

In the present study, only tumour DNA was available for genotyping and this introduces a risk for misclassification of genotypes because loss of heterozygosity and gene mutations frequently occur at several sites during carcinogenesis. However, breast tumour tissues are a mixture of stromal, inflammatory and malignant cells, and genetic lesions usually affect only a subgroup of tumour cells. Therefore the presence of normal cells and malignant cells without DNA lesions in the locus of *CYP3A5*, *CYP2D6*, *SULT1A1 *and *UGT2B15 *decreases the risk of genotype misclassification. This is also supported by the fact that allele frequencies of CYP3A5*3, CYP2D6*1, SULT1A1*2 and UGT2B15*2 in the current study population are comparable to those reported by others in Caucasian populations [18,19,26,27].

## Conclusion

Taken together, the current investigation and earlier published reports [25,27,35] of randomised patients connected with polymorphisms in metabolic enzymes and the outcome of tamoxifen give rise to questions about the hypothesis that genotypes contributing to the biosynthesis of the ER-active metabolites (such as endoxifen and 4-hydroxy-tamoxifen) improve the benefit of tamoxifen. Investigations by Nowell's group [35] and from our laboratory indicate that patients who carry the SULT1A1*1, CYP2D6*4 and CYP3A5*3 alleles, which give less ER-active metabolites, might benefit from tamoxifen [25,27], whereas others have found the opposite, particularly regarding CYP2D6 [28]. However, the metabolism of tamoxifen is complex and the enzymes contributing in this process can also be affected by co-prescribed drugs. The mechanisms responsible for the resistance are therefore unlikely to be explained by a single polymorphism but rather by a combination of several mechanisms. Nevertheless, the present investigation suggests that the genotype of *CYP3A5 *may contribute to tamoxifen response.

## Abbreviations

CI = confidence interval; CYP3A4 = cytochrome P450 3A4; CYP3A5 = cytochrome P450 3A5; CYP2D6 = cytochrome P450 2D6; ER = oestrogen receptor; HR = hazard ratio; PCR = polymerase chain reaction; RFS = recurrence-free survival; SSRI = selective serotonin reuptake inhibitors; SULT1A1 = sulphotransferase 1A1; TEAA = triethylammonium acetate; UGT2B15 = UDP-glucuronosyltransferase 2B15.

## Competing interests

The authors declare that they have no competing interests.

## Authors' contributions

PW conducted part of the laboratory work, performed the statistical analyses and drafted the manuscript. SE conducted part of the laboratory work. JC conceived the statistical analyses. OS contributed with the coordination of the study and participated in the preparation of the manuscript. BN provided tumour material and clinical data. SW conceived the study and participated in its design and coordination. All authors participated in the preparation of the manuscript and approved the final version.

## Figures and Tables

**Table 1:** Use of χ^2 ^test to compare genotype and tumour characteristics in ER-positive patients with breast cancer

Genotype	Characteristics							
	Tumour size (mm)^a^			Tumour stage		Nodal involvement		
	≤20	21–50	>50	II	III	0	1–3	>4
*CYP3A5 *(*n *= 663)	*n *(%)			*n *(%)		*n *(%)		
*1/*1	1 (14.3)	5 (71.4)	1 (14.3)	6 (85.7)	1 (14.3)	3 (42.9)	3 (42.9)	1 (14.3)
*1/*3	28 (30.4)	59 (64.1)	5 (5.4)	83 (89.2)	10 (10.8)	27 (29.0)	45 (48.4)	21 (22.6)
*3/*3	150 (26.8)	369 (66.0)	40 (7.2)	479 (85.1)	84 (14.9)	177 (31.4)	220 (39.1)	166 (29.5)
*P*	0.78			0.57		0.40		
*CYP2D6 *(*n *= 677)								
*1/*1	132 (28.9)	294 (64.3)	31 (6.8)	393 (85.4)	67 (14.6)	142 (30.9)	194 (42.2)	124 (27.0)
*1/*4	45 (24.7)	125 (66.7)	12 (6.6)	159 (86.9)	24 (13.1)	56 (30.6)	73 (39.9)	54 (29.5)
*4/*4	9 (27.3)	21 (63.6)	3 (9.1)	29 (85.3)	5 (14.7)	10 (29.4)	14 (41.2)	10 (29.4)
*P*	0.83			0.89		0.98		
*SULT1A1 *(*n *= 677)								
*1/*1	77 (30.5)	163 (64.7)	12 (4.8)	223 (88.1)	30 (11.9)	80 (31.6)	104 (41.1)	69 (27.3)
*1/*2	79 (25.0)	209 (66.1)	28 (8.9)	269 (84.3)	50 (15.7)	85 (26.6)	142 (44.5)	92 (28.8)
*2/*2	30 (28.8)	68 (65.4)	6 (5.8)	89 (84.8)	16 (15.2)	43 (41.0)	35 (33.3)	27 (25.7)
*P*	0.26			0.41		0.09		
*UGT2B15 *(*n *= 445)								
*1/*1	38 (31.6)	71 (59.2)	11 (9.2)	99 (82.5)	21 (17.5)	41 (34.2)	41 (34.2)	38 (31.6)
*1/*2	46 (24.9)	129 (69.7)	10 (5.4)	160 (85.6)	27 (14.4)	58 (31.0)	75 (40.1)	54 (28.9)
*2/*2	40 (29.2)	82 (59.9)	15 (10.9)	110 (79.7)	28 (20.3)	37 (26.8)	68 (49.3)	33 (23.9)
*P*	0.18			0.38		0.18		

Results are shown as *n *(%). ER, oestrogen receptor. ^a^Information on tumour size was missing for five patients.

**Table 2:** Hazard ratio (HR) between genotypes and randomisation calculated with the Cox proportional-hazard model

Genotype	Duration of tamoxifen treatment					
	2 years			5 years		
	HR (*n*)	95% CI	*P*	HR (*n*)	95% CI	*P*
CYP3A5*1/*1, *1/*3	1.00 (16)			1.00 (13)		
CYP3A5*3/*3	2.84 (83)	0.68–11.99	*0.15*	0.20 (95)	0.07–0.55	*0.002*
CYP2D6*1/*1	1.00 (72)			1.00 (79)		
CYP2D6*1/*4, *4/*4	0.87 (31)	0.38–1.97	*0.74*	0.33 (32)	0.08–1.43	*0.14*
SULT1A1*1/*2, *2/*2	1.00 (71)			1.00 (73)		
SULT1A1*1/*1	0.33 (32)	0.12–0.96	*0.04*	0.83 (36)	0.29–2.36	*0.73*
UGT2B15*1/*1	1.00 (17)			1.00 (26)		
UGT2B15*1/*2, *2/*2	1.18 (62)	0.39–3.52	*0.77*	1.94 (55)	0.53–7.06	*0.31*

Cases with less than 2 years of follow-up time were excluded. For each randomisation, patients with the proposed 'low-risk' alleles were used as reference. CI, confidence interval.

## References

[B1] Early Breast Cancer Trialist's Collaborative Group (EBCTCG) (1998). Tamoxifen for early breast cancer: an overview of randomised trials. Lancet.

[B2] Stearns V, Johnson M, Rae JM, Morocho A, Novielli A, Bhargava P, Hayes DF, Desta Z, Flockhart DA (2003). Active tamoxifen metabolite plasma concentrations after coadministration of tamoxifen and the selective serotonin reuptake inhibitor paroxetine. J Natl Cancer Inst.

[B3] Johnson MD, Zuo H, Lee K-H, Trebley JP, Rae JM, Weatherman RV, Desta Z, Flockhart DA, Skaar TC (2004). Pharmacological characterization of 4-hydroxy-N-desmethyl tamoxifen, a novel active metabolite of tamoxifen. Breast Cancer Res Treat.

[B4] Lim YC, Desta Z, Flockhart DA, Skaar T (2005). Endoxifen (4-hydroxy-*N*-desmethyl-tamoxifen) has anti-estrogenic effects in breast cancer cells with potency similar to 4-hydroxy-tamoxifen. Cancer Chemother Pharmacol.

[B5] MacCallum J, Cummings J, Dixon JM, Miller WR (2000). Concentrations of tamoxifen and its major metabolites in hormone responsive and resistant breast tumours. Br J Cancer.

[B6] Kisanga ER, Gjerde J, Guerrieri-Gonzaga A, Pigatto F, Pesci-Feltri A, Robertson C, Serrano D, Pelosi G, Decensi A, Lien EA (2004). Tamoxifen and metabolite concentrations in serum and breast cancer tissue during three dose regimens in a randomized preoperative trial. Clin Cancer Res.

[B7] Fabian C, Tilzer L, Sternson L (1981). Comparative binding affinities of tamoxifen, 4-hydroxytamoxifen, and desmethyltamoxifen for estrogen receptors isolated from human breast carcinoma: correlation with blood levels in patients with metastatic breast cancer. Biopharm Drug Dispos.

[B8] Coezy E, Borgna J-L, Rochefort H (1982). Tamoxifen and metabolites in MCF7 cells: correlation between binding to estrogen receptor and inhibition of cell growth. Cancer Res.

[B9] Murphy CS, Langan-Fahey SM, McCague R, Jordan VC (1990). Structure function relationship of hydroxylated metabolites of tamoxifen that control the proliferation of estrogen-responsive T47D breast cancer cells in vitro. Mol Pharmacol.

[B10] Jacolot F, Simon I, Dreano Y, Beaune P, Riche C, Berthou F (1991). Identification of the cytochrome P450 IIIA family as the enzymes involved in the N-demethylation of tamoxifen in human liver microsomes. Biochem Pharmacol.

[B11] Crewe HK, Ellis SW, Lennard MS, Tucker GT (1997). Variable contribution of cytochromes P450 2D6, 2C9 and 3A4 to the 4-hydroxylation of tamoxifen by human liver microsomes. Biochem Pharmacol.

[B12] Dehal SS, Kupfer D (1997). CYP2D6 catalyses tamoxifen 4-hydroxylation in human liver. Cancer Res.

[B13] Jordan VC, Haldemann B, Allen KE (1981). Geometric isomers of substituted triphenylethylenes and antiestrogen action. Endocrinology.

[B14] Katzenellenbogen BS, Norman MJ, Eckert RL, Peltz SW, Mangel WF (1984). Bioactivities, estrogen receptor interactions, and plasminogen activator-inducing activities of tamoxifen and hydroxytamoxifen isomers in MCF-7 human breast cancer cells. Cancer Res.

[B15] Malet C, Spritzer P, Cumins C, Guillaumin D, Mauvais-Jarvis P, Kuttenn F (2002). Effect of 4-hydroxytamoxifen isomers on growth and ultrastructural aspects on normal human breast epithelial (HBE) cells in culture. J Steroid Biochem Mol Biol.

[B16] Osborne CK, Wiebe VJ, McGuire WL, Ciocca DR, DeGregorio MW (1992). Tamoxifen and the isomers of 4-hydroxytamoxifen in tamoxifen-resistant tumors from breast cancer patients. J Clin Oncol.

[B17] Nishiyama T, Ogura K, Nakano H, Ohnuma T, Kaku T, Hiratsuka A, Muro K, Watabe T (2002). Reverse geometrical selectivity in glucuronidation and sulfation of *cis*- and *trans*-4-hydroxytamoxifens by human liver UDP-glucuronosyltransferases and sulfotransferases. Biochem Pharmacol.

[B18] Kuehl P, Zhang J, Lin Y, Lamba J, Assem M, Shuetz J, Watkins PB, Daly A, Wrighton SA, Hall SD (2001). Sequence diversity in *CYP3A *promoters and characterization of the genetic basis of polymorphic CYP3A5 expression. Nat Genet.

[B19] Hustert E, Haberl M, Burk O, Wolbold R, He YQ, Klein K, Nuessler AC, Neuhaus P, Klattig J, Eiselt R (2001). The genetic determinants of CYP3A5 polymorphism. Pharmacogenetics.

[B20] Lamba JK, Lin YS, Schuetz EG, Thummel KE (2002). Genetic contribution to variable human CYP3A-mediated metabolism. Adv Drug Deliv Rev.

[B21] Hanioka N, Kimura S, Meyer UA, Gonzalez FJ (1990). The human *CYP2D *locus associated with a common genetic defect in drug oxidation: a G1934----A base change in intron 3 of a mutant *CYP2D6 *allele results in an aberrant 3' splice recognition site. Am J Hum Genet.

[B22] Raftogianis RB, Wood TC, Otterness DM, Van Loon JA, Weinshilboum RM (1997). Phenol sulfotransferase pharmacogenetics in humans: association of common *SULT1A1 *alleles with TS PST phenotype. Biochem Biophys Res Commun.

[B23] Iida A, Saito S, Sekine A, Mishima C, Kitamura Y, Kondo K, Harigae S, Osawa S, Nakamura Y (2002). Catalog of 86 single-nucleotide polymorphisms (SNPs) in three uridine diphosphate glycosyltransferase genes: *UGT2A1*, *UGT2B15*, and *UGT8*. J Hum Genet.

[B24] Lévesque E, Beaulieu M, Green MD, Tephly TR, Bélanger A, Hum DW (1997). Isolation and characterization of UGT2B15(Y^85^): a UDP-glucuronosyltransferase encoded by a polymorphic gene. Pharmacogenetics.

[B25] Wegman P, Vainikka L, Stål O, Nordenskjöld B, Skoog L, Rutqvist LE, Wingren S (2005). Genotype of metabolic enzymes and the benefit of tamoxifen in postmenopausal breast cancer patients. Breast Cancer Res.

[B26] Coughtrie MW, Gilissen RA, Shek B, Strange RC, Fryer AA, Jones PW, Bamber DE (1999). Phenol sulphotransferase SULT1A1 polymorphism: molecular diagnosis and allele frequencies in Caucasian and African populations. Biochem J.

[B27] Nowell SA, Ahn J, Rae JM, Scheys JO, Trovato A, Sweeney C, MacLeod SL, Kadlubar FF, Ambrosone CB (2005). Associtation of genetic variation in tamoxifen-metabolizing enzymes with overall survival and recurrence of disease in breast cancer patients. Breast Cancer Res Treat.

[B28] Goetz MP, Rae JM, Suman VJ, Safgren SL, Ames MM, Visscher DW, Reynolds C, Couch FJ, Lingle WL, Flockhart DA (2005). Pharmacogenetics of tamoxifen biotransformation is associated with clinical outcomes of efficacy and hot flashes. J Clin Oncol.

[B29] Tucker AN, Tkaczuk KA, Lewis LM, Tomic D, Lim CK, Flaws JA (2005). Polymorphisms in cytochrome P4503A5 (CYP3A5) may be associated with race and tumor characteristics, but not metabolism and side effects of tamoxifen in breast cancer patients. Cancer Lett.

[B30] Jin Y, Desta Z, Stearns V, Ward B, Ho H, Lee KY, Skaar T, Storniolo AM, Li L, Araba A (2005). CYP2D6 genotype, antidepressant use, and tamoxifen metabolism during adjuvant breast cancer treatment. J Natl Cancer Inst.

[B31] Koch I, Weil R, Wolbold R, Brockmöller J, Husert E, Burk O, Nuessler A, Neuhaus P, Eichelbaum M, Zanger U (2002). Interindividual variability and tissue-specificity in the expression of cytochrome P450 3A mRNA. Drug Metab Dispos.

[B32] Shih PS, Huang JD (2002). Pharmacokinetics of midazolam and 1'-hydroxymidazolam in Chinese with different *CYP3A5 *genotypes. Drug Metab Dispos.

[B33] Westlind-Johnsson A, Malmebo S, Johansson A, Otter C, Andersson TB, Johansson I, Edwards RJ, Boobis AR, Ingelman-Sundberg M (2003). Comparative analysis of CYP3A expression in human liver suggests only a minor role for CYP3A5 in drug metabolism. Drug Metab Dispos.

[B34] van Schaik RHN (2005). Cancer treatment and pharmacogenetics of cytochrome P450 enzymes. Invest New Drugs.

[B35] Nowell S, Sweeney C, Winters M, Stone A, Lang NP, Hutchins LF, Kadlubar FF, Ambrosone CB (2002). Association between sulfotransferase 1A1 genotype and survival of breast cancer patients receiving tamoxifen therapy. J Natl Cancer Inst.

[B36] Lehmann D, Nelsen J, Ramanath V, Newman N, Duggan D, Smith A (2004). Lack of attenuation in the antitumor effect of tamoxifen by chronic CYP isoform inhibition. J Clin Pharmacol.

[B37] Gallicchio L, Tkaczuk K, Lord G, Danton M, Lewis LM, Lim CK, Flaws JA (2004). Medication use, tamoxifen (TAM), and TAM metabolite concentrations in women with breast cancer. Cancer Lett.

